# Frequently increased epidermal growth factor receptor (EGFR) copy numbers and decreased BRCA1 mRNA expression in Japanese triple-negative breast cancers

**DOI:** 10.1186/1471-2407-8-309

**Published:** 2008-10-25

**Authors:** Tatsuya Toyama, Hiroko Yamashita, Naoto Kondo, Katsuhiro Okuda, Satoru Takahashi, Hidefumi Sasaki, Hiroshi Sugiura, Hirotaka Iwase, Yoshitaka Fujii

**Affiliations:** 1 Department of Oncology, Immunology and Surgery, Nagoya City University Graduate School of Medical Sciences, 1 Kawasumi, Mizuho-cho, Mizuho-ku, Nagoya 467-8601, Japan; 2 Department of Experimental Pathology and Tumor Biology, Nagoya City University Graduate School of Medical Sciences, 1 Kawasumi, Mizuho-cho, Mizuho-ku, Nagoya 467-8601, Japan; 3 Division of Pathology, Nagoya City University Hospital, 1 Kawasumi, Mizuho-cho, Mizuho-ku, Nagoya 467-8601, Japan; 4 Department of Breast and Endocrine Surgery, Kumamoto University Graduate School of Medical Sciences, 1-1-1 Honjo, Kumamoto 860-8556, Japan

## Abstract

**Background:**

Triple-negative breast cancer (estrogen receptor-, progesterone receptor-, and HER2-negative) (TNBC) is a high risk breast cancer that lacks specific therapy targeting these proteins.

**Methods:**

We studied 969 consecutive Japanese patients diagnosed with invasive breast cancer from January 1981 to December 2003, and selected TNBCs based on the immunohistochemical data. Analyses of epidermal growth factor receptor (*EGFR*) gene mutations and amplification, and *BRCA*1 mRNA expression were performed on these samples using TaqMan PCR assays. The prognostic significance of TNBCs was also explored. Median follow-up was 8.3 years.

**Results:**

A total of 110 (11.3%) patients had TNBCs in our series. Genotyping of the *EGFR *gene was performed to detect 14 known *EGFR *mutations, but none was identified. However, *EGFR *gene copy number was increased in 21% of TNBCs, while only 2% of ER- and PgR-positive, HER2-negative tumors showed slightly increased *EGFR *gene copy numbers. Thirty-one percent of TNBCs stained positive for EGFR protein by immunohistochemistry. *BRCA1 *mRNA expression was also decreased in TNBCs compared with controls. Triple negativity was significantly associated with grade 3 tumors, TP53 protein accumulation, and high Ki67 expression. TNBC patients had shorter disease-free survival than non-TNBC in node-negative breast cancers.

**Conclusion:**

TNBCs have an aggressive clinical course, and *EGFR *and *BRCA1 *might be candidate therapeutic targets in this disease.

## Background

Although the overall mortality due to breast cancer in the United States has decreased, attributed in part to breast screening and early application of various treatments, overall mortality in Japan is still increasing [1]. Breast cancers represent a heterogeneous group of tumors that are diverse in behavior, outcome, and sensitivity to therapy. To reduce mortality from breast cancer, it would be desirable to identify and characterize tumors having a poor prognosis. Emerging data demonstrate that stratification of tumors by gene expression profiles divides breast cancer into four common subtypes which are associated with different clinical outcome [2]. Two of them are estrogen receptor (ER)-positive (luminal and luminal/HER2+) and two are ER-negative (basal-like and HER2 positive) [2,3].

Although the immunohistochemical staining profile can be a useful surrogate for gene expression analysis, the optimum immunohistochemical profile of the basal-like subtype remains unclear. However, the basal-like category is composed almost entirely of triple-negative breast cancers (TNBCs) (i.e., tumors lacking ER, progesterone receptor (PgR) and HER2 expression) [3-5]. A simplified method of classification, based on immunohistochemical assays for ER, PgR, and HER2, is clinically useful, and clinicians are increasingly taking triple-negative status into account in clinical decision-making and therapeutic protocol design. Epidermal growth factor receptor (EGFR) is more frequently expressed in TNBCs than in non-TNBCs [6,7]. Therefore, the EGFR is considered a candidate treatment target for TNBCs.

Cluster analyses of microarray expression profiling data have shown that familial *BRCA1 *mutant tumors tend to fall into the basal-like category [2]. Some studies have indicated that *BRCA1 *mRNA expression is lower in basal-like sporadic cancers [8], and in approximately 30% of sporadic breast cancers [9]. It is believed that this may be the result of an epigenetic mechanism acting on the *BRCA1 *gene or a dysfunction in the upstream pathways regulating *BRCA1 *expression [8,10]. These sporadic tumors with reduced *BRCA*1 expression tend to be triple-negative, and remarkably similar to *BRCA1*-mutated tumors [8].

Here, we present data on a large series of TNBCs with long-term follow-up and data on the status of their *EGFR *and *BRCA1 *genes.

## Methods

### Patients

A total of 969 surgically resected breast carcinomas with tissue blocks available were selected from the archive of the Department of Breast and Endocrine Surgery, Nagoya City University Hospital in Japan. Specimens were obtained from patients who underwent surgery between January 1981 and December 2003. Tissues were fixed in 10% buffered formalin and embedded in paraffin or placed in liquid nitrogen immediately after resection and stored at -80°C until DNA and RNA extraction. Informed consent was obtained before the surgery. The histological grade was estimated according to the Bloom and Richardson method proposed by Elston and Ellis [11]. Analysis of clinical outcome was performed in a total of 557 consecutive breast cancer patients operated from January 1981 to December 1999, because their clinicopathological and survival data were available for this study. Disease-free survival (DFS) was defined as the interval from the date of primary surgery to the first locoregional recurrence or distant metastasis. The distant disease-free survival (DDFS) was defined as the interval from the date of primary surgery to the identification of distant metastasis. The median follow-up period was 8.3 years (range 3 to 281 months). Relapse data were available in 543 of 557 patients examined: 159 (28.5%) experienced disease recurrence; 99 (17.7%) showed distant relapse; and 75 (13.4%) had died. This protocol was approved by the Institutional Review Board of Nagoya City University Graduate School of Medical Sciences.

### DNA and RNA extraction

Genomic DNA and total RNA from homogeneous microscopically confirmed breast cancer tissues was isolated from approximately 500 mg of each frozen specimen using a phenol-chloroform extraction method for DNA extraction, and with TRIZOL reagent (Invitrogen Japan K.K., Tokyo, Japan) for RNA extraction according to the manufacturer's recommendations. Total RNA was also isolated from MCF-7 cells with TRIZOL reagent (Invitrogen).

### Immunohistochemical analysis

One 4-μm section from each paraffin block specimen was first stained with hematoxylin and eosin in order to ascertain that an adequate number of invasive ductal carcinoma cells were present and that quality of fixation was adequate for immunohistochemical analysis. Serial sections (4-μm) were then prepared from suitable blocks and float-mounted on adhesive-coated glass slides for ERα, PgR, HER2, TP53, Ki67, cytokeratin (CK) 5/6, CK14 and EGFR staining. Primary antibodies were mouse monoclonal anti-human antibodies against ERα (1D5; DAKO, Glostrup, Denmark) at a 1:100 dilution, PgR (PgR636; DAKO) at a 1:100 dilution, TP53 (PAb1801; Novocastra, Newcastle, UK) at 1:50, Ki67 (MIB-1; DAKO) at 1:100, CK5/6 (D5/16 B4; DAKO) at 1:50 and CK14 (LL002; NeoMarkers, Fremont, CA) at 1:200. Rabbit anti-human c-erbB2 oncoprotein antibody (DAKO) at a 1:200 dilution was used for HER2. The DAKO EnVision system (DAKO EnVision-labeled polymer, peroxidase) was used for detection of ERα, PgR, HER2, Ki67, CK5/6, and CK14. The streptavidin-biotin system (SAB-PO kit; Nichirei Co., Inc., Tokyo, Japan) was applied for detection of the bound TP53 antibody. Immunostaining for EGFR was performed using the EGFRpharmDx assay detection system (prediluted; DAKO).

### Interpretation of immunohistochemical staining

Immunostained specimens were scored after the entire slide had been evaluated by light microscopy. The expression of ERα was scored by assigning a proportion score and an intensity score according to Allred's procedure [12]. Any brown nuclear staining in invasive breast epithelium was counted toward the proportion score. Tumors with scores of 3 or greater were considered to be positive for ERα expression. HER2 immunostaining was evaluated using the same method as is employed by the HercepTest (DAKO). To determine the score of HER2 expression the membrane staining pattern was estimated and scored on a scale of 0 to 3+. Tumors with scores of 0 and 1 were considered to be negative, and tumors with a score of 3 were considered to be positive for HER2 overexpression. All tumors with a score of 2 were omitted from the analysis in this study because fluorescent *in situ *hybridization (FISH) analysis was not carried out on them. In the case of TP53, tumors were considered immunohistochemically positive when ≥ 10% of the tumor cells showed nuclear staining [13,14]. The expression status of Ki67 was assessed according to the estimated proportion of nuclear staining of tumor cells that were positively stained. Scoring criteria for Ki67 were as follows (in the form proportion of nuclear staining = score): none = 0, <1/100 = 1, 1/100-1/10 = 2, 1/10-1/2 = 3, and >1/2 = 4 [15]. Tumors with a score of 3 or greater for Ki67 were considered to have high Ki67 expression. Positivity for CK5/6 and CK14 was defined as the detection of more than 5% of invasive tumor cells showing strong cytoplasmic and membrane staining [16]. Immunostaining for EGFR was interpreted as positive when at least 10% of tumor cells showed strong membrane staining [7,17].

### Genetic analysis of EGFR

The primers and TaqMan probes were designed for 14 known mutations in the *EGFR *gene [18]. TaqMan genotyping assays for *EGFR *mutations were performed on Applied Biosystems 7500 Fast Real-Time PCR equipment (Applied Biosystems) as described previously [18]. The results were analyzed using the allelic discrimination assay program [18].

### Real-Time Reverse Transcription (RT)-PCR

RT was performed as described previously [22]. Real-time quantitative RT-PCR of the *BRCA1 *gene was performed using TaqMan Universal PCR Master Mix (Applied Biosystems) in the Applied Biosystems 7500 Fast Real-Time PCR System, following the manufacturer's instructions. The primers and probes for the *BRCA1 *mRNA expression assay are commercially available (TaqMan Gene Expression Assays, Assay ID: Hs00173233_m1, Applied Biosystems). The PCR conditions were as follows: initial denaturation at 95°C for 2 sec, followed by 40 cycles of 95°C for 3 sec, and 60°C for 30 sec. To ensure the fidelity of mRNA extraction and reverse transcription, *BRCA1 *signals from all samples were normalized against *GAPDH *as an internal control. The primers and probes for *GAPDH *mRNA expression assay are commercially available (TaqMan Gene Expression Assays, Assay ID: Hs99999905_m1, Applied Biosystems). The PCR conditions for amplification of *GAPDH *were as follows: initial denaturation at 95°C for 2 sec, followed by 40 cycles of 95°C for 3 sec, and 60°C for 30 sec. For each PCR run, a standard curve was constructed with serial dilutions of cDNA obtained from MCF-7 cells, which express *BRCA1 *mRNA.

### Statistical analyses

All molecular and immunohistochemical analyses were performed blinded to clinical data. Statistical calculations were performed with StatView-J 5.0 software (SAS Institute Inc., Cary, NC). The relationship between the frequency of TNBC or non-TNBC and clinicopathologic factors were assessed by χ^2 ^and Fisher's exact probability tests. The Mann-Whitney U-test was performed for the analyses of the *EGFR *gene copy number and *BRCA1 *mRNA expression. DFS and DDFS curves were generated by the Kaplan-Meier method and verified by the log-rank test. Cox proportional hazards regression analysis was used for univariate and multivariate analyses of prognostic values. Differences were considered significant when a *P *value < 0.05 was obtained.

## Results

### Patient Characteristics and Immunohistochemical results

In total, 969 Japanese cases of invasive breast cancer undergoing surgery at Nagoya City University Hospital from January 1981 to December 2003 were informative for the 3 markers ER, PgR, and HER2. Of these informative cases, 110 (11.3%) were found to be TNBCs. Immunohistological analyses of TP53 and Ki67, and clinicopathological analysis were performed in 531 consecutive patients from 1981 to 1999. Table 1 shows the features of 71 TNBCs compared with 415 non-TNBCs concerning different clinicopathological variables and biomarkers. All tumors with a HER2 score 2+ were omitted from this analysis, as indicated in Methods. TNBC was significantly associated with grade 3 tumors (P < 0.0001), with TP53 protein accumulation (P < 0.0001) and with high Ki67 expression (P < 0.0001), as well as lymph node status (P = 0.02).

**Table 1:** Characteristics of Patients with Different Tumor Subtypes

**Variables**	**Total**		**Triple negative**		**Non-triple negative**		**P value**
		
	***N = 486 (100%)***		***N = 71 (15%)***		***N = 415 (85%)***		
	***No.***	***%***	***No.***	***%***	***No.***	***%***	
Age, years							0.49
≤50	198	41	32	44	166	40	
>50	288	59	39	56	249	60	
Size							0.23
≤2 cm	181	37	21	30	160	39	
>2 cm	288	59	46	65	242	58	
Unknown	17		4	5	13	3	
Nodal status							0.02
Negative	275	57	48	68	227	55	
Positive	192	40	19	27	173	42	
Unknown	19	3	4	5	15	3	
Histology							0.14
Ductal	454	94	66	93	388	93	
Lobular	16	3	0	0	16	4	
Other	16	3	5	7	11	3	
Grade							<0.0001
1	92	19	4	6	88	21	
2	273	56	32	45	241	58	
3	101	21	34	48	67	16	
Unknown	20	4	1	1	19	5	
TP53 expression							<0.0001
Negative	392	81	43	61	349	84	
Positive	92	19	28	39	64	15	
Unknown	2	0	0	0	2	1	
Ki67 expression							<0.0001
Low	333	69	34	48	299	72	
High	150	31	37	52	113	27	
Unknown	3	1	0	0	3	1	

Next, immunohistological analyses of EGFR, CK 5/6, and CK14 were performed for all TNBCs available in this study, operated from January 1981 to December 2003. As shown in Table 2, 31% (33 cases) of TNBC were positive for EGFR protein, 52% (57 cases) expressed CK5/6, and 55% (61 cases) CK14. A total of 77 (70%) had a basal-like phenotype according to Nielsen's definition (TNBC with CK5/6 and/or EGFR expression) [3,4].

**Table 2:** Immunohistochemical Findings in Triple Negative Breast Cancer

**Variables**	**Triple negative (N = 110)**	
	
	***No.***	***%***
***EGFR***		
Negative	69	62
Positive	33	31
Unknown	8	7
***Cytokeratin 5/6***		
Negative	47	43
Positive	57	52
Unknown	6	5
***Cytokeratin 14***		
Negative	43	40
Positive	61	55
Unknown	6	5
***Cytokeratin 5/6/14***		
Negative	32	30
Posotive	72	65
Unknown	6	5
***Basal-like phenotype***		
Negative	24	22
Positive	77	70
Unknown	9	8

### Genomic analysis of *EGFR*

**Figure 1:**
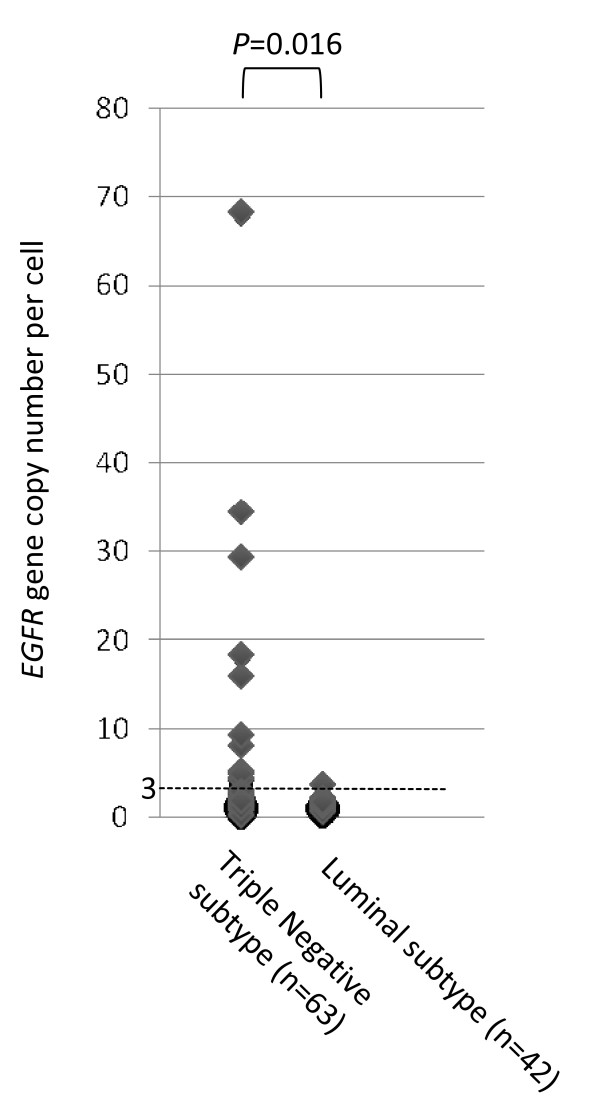
Epidermal growth factor receptor (*EGFR*) gene copy number in triple-negative and luminal subtypes in Japanese breast cancer patients.

We sought mutations in the *EGFR *gene in TNBCs, because *EGFR *mutations were found in a subset of Japanese non-small lung cancers, despite being very rare in Caucasians [23], and because these mutations were suggested to predict responses to gefitinib therapy [23,24]. The sequences of primers and probes in TaqMan genotyping assays for the EGFR gene are shown in Table 3. Genomic DNA was available in 63 of 110 TNBC tissues, and genotyping could be successfully performed for the 14 known *EGFR *mutations in 58 of these. However, none of the 58 TNBC showed any known *EGFR *mutations. We previously reported an increased *EGFR *copy number in a subset of Japanese non-small lung cancers [18]. As 31% of TNBCs were positive for EGFR protein in this series, we measured *EGFR *gene copy number in 63 TNBCs compared with 42 ER and/or PgR-positive but HER2-negative consecutive breast cancers, (which are also termed luminal subtype [2]). It was found that 21% (12 cases) of TNBCs contained an increased *EGFR *gene copy number while only one (2%) of luminal subtype tumors showed slightly increased *EGFR *gene copy (Mann-Whitney U-test: *P *= 0.016, Fig. 1). We then sought any correlations between the *EGFR *gene copy number and EGFR protein expression based on the immunohistochemical analysis in TNBCs. Only 2 of 14 TNBCs showing EGFR protein expression demonstrated increased *EGFR *gene copy number (Table 4). There were no significant correlations between these parameters.

**Table 3:** Sequence of the mutation-specific TaqMan probes and PCR primers for the *EGFR *gene

**Mutation No.**	**Probe name**	**Nucleotide**	**Amino Acid**	**Primer sequence (forward)**
	WT1			CCCAGAAGGTGAGAAAGTTAAAATTC
1	Del 1a	2235-2249del	E746-A750del	(same as above)
2	Del 1b	2236-2250del		(same as above)
3	Del 2	2254-2277del	S752-I759del	(same as above)
	WT2			(same as above)
4	Del 3	2239-2247del, 2248G>C	L747-E749del, A750P	(same as above)
5	Del 4	2240-2257del	L747-S752del, P753S	(same as above)
6	Del 5	2238-2255del, 2237A>T	L747-A750del, E746V	(same as above)
7	Del 6	2240-2251del	L747-A750del, T747S	(same as above)
	WT3			TGAGGATCTTGAAGGAAACTGAATTC
8	G719C	2155G>T	G719C	(same as above)
9	G719S	2155G>A	G719S	(same as above)
	WT4			CCGCAGCATGTCAAGATCAC
10	L858R	2573T>G	L858R	(same as above)
	WT5			(same as above)
11	L861Q	2582T>A	L861Q	(same as above)
	WT6			CCCAGAAGGTGAGAAAGTTAAAATTC
12	L747Del	2239-2262del	L747-K754del	(same as above)
13	N756Ins	2265-2267InsCAA	N756Ins	(same as above)
	WT7			GGAGGACCGTCGCTTGGT
14	L838P	2513T>C	L838P	(same as above)

**TaqMan probe**	**Primer sequence (reverse)**			

VIC-ATTAAGAGAAGCAACATCT	CCCACACAGCAAAGCAGAAA			
FAM-CGCTATCAAAACATCT	(same as above)			
FAM-CTATCAAGACATCTCC	(same as above)			
FAM-AGAAGCAACACTCGAT	(same as above)			
VIC-CGAAAGCCAACAAG	(same as above)			
FAM-CAAGGAACCAACATC	(same as above)			
FAM-AAGGAATCGAAAGCC	(same as above)			
FAM-CAAGGTTCCGAAAGC	(same as above)			
FAM-TCAAGGAATCATCTCC	(same as above)			
VIC-AAGTGCTGGGCTCC	TGCCAGGGACCTTACCTTATACA			
FAM-AAAGTGCTGTGCTCC	(same as above)			
FAM-AAAGTGCTGTGCTCC	(same as above)			
VIC-TTGGGCTGGCCAAA	TCCTTCTGCATGGTATTCTTTCTCT			
FAM-TTGGGCGGGCCAA	(same as above)			
VIC-CCAAACTGCTGGGTG	(same as above)			
FAM-CCAAACAGCTGGGTG	(same as above)			
VIC-ATTAAGAGAAGCAACATCT	CCCACACAGCAAAGCAGAAA			
FAM-CTATCAAGGAAGCCAACAA-MGB	(same as above)			
FAM-CCAACAACAAGGAAAT-MGB	(same as above)			
VIC-CGCGACCTGGCAG-MGB	CCCAAAATCTGTGATCTTGACATG			
FAM-CGCGACCCGGCAG-MGB	(same as above)			

*EGFR *gene copy numbers in breast cancer tissues were estimated with 7500 Fast Real-Time PCR using a QuantiTect SYBR Green Kit (Qiagen Inc, Valencia, CA) as described previously [18]. Briefly, we have quantified each tumor DNA by comparing the target locus to the reference *Line-1*, a repetitive element for which copy numbers per haploid genome are similar among all normal and neoplastic human cells [19,20]. The relative *EGFR *copy number level was normalized to normal human genomic DNA as the calibrator. Copy number changes relative to the *Line-1 *gene and the calibrator were determined by the formula (T_EGFR_/T_Line-1_)/(C_EGFR_/C_Line-1_), where T_EGFR_/T_Line-1 _are quantity from tumor DNA by using *EGFR *and *Line-1*, and C_EGFR_/C_Line-1 _are quantity from calibrator by using *EGFR *and *Line-1 *[19,20]. Primer sequences for the *EGFR *gene used in this study were: forward, CCACCAAATTAGCCTGGACA; reverse, CGCGACCCTTAGGTATTCTG. *EGFR *gene copy number > 3 was defined as positive [21].

**Table 4:** Relationship between *EGFR *Copy Number and the Immunohistochemical staining of EGFR protein in Triple Negative Breast Cancer

	**Increased EGFR gene copy No.**	**No increased EGFR gene copy No.**
***EGFR IHC***		
0	4	14
1	6	22
2	2	8
3	0	2

IHC, immnohistochemistry

### *BRCA1 *mRNA expression analysis

**Figure 2:**
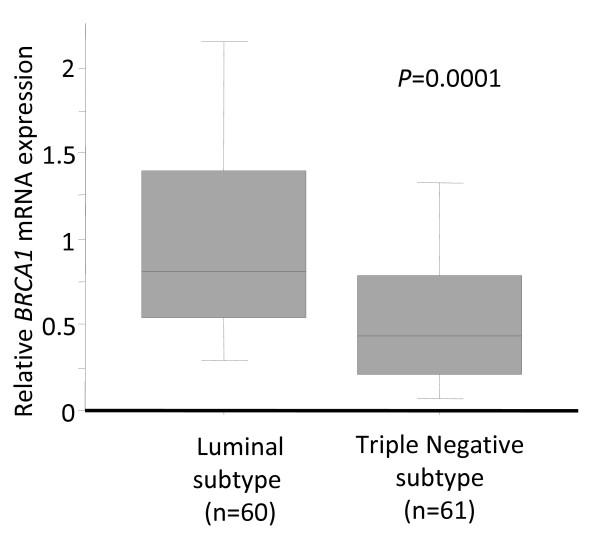
***BRCA1*****mRNAexpression in triple-negative and luminal subtypes in Japanese breast cancer patients**.

Loss of BRCA1 function has been found in basal-like breast cancer. Therefore, we assessed *BRCA1 *mRNA expression in 61 TNBC and 60 consecutive luminal subtype breast cancers. As shown in Fig. 2, *BRCA1 *mRNA expression was significantly decreased in TNBCs compared to luminal subtype breast cancers. *BRCA1 *gene mutations were not analyzed in this study. Therefore, TNBCs in our series may include both *BRCA*1-mutant and sporadic TNBCs. Nonetheless, the fact remains that significantly reduced *BRCA1 *mRNA expression was observed in these Japanese TNBCs.

### Clinical outcome by treatment epoch

**Figure 3:**
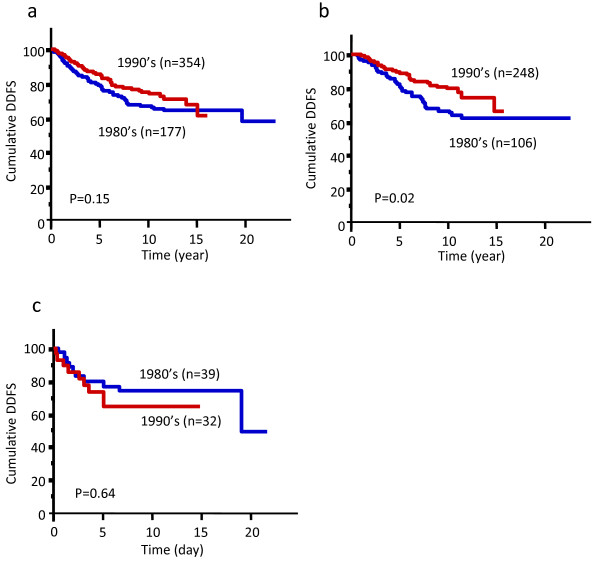
**Distant disease-free survival (DDFS) according to the treatment epoch.** (a) all patients, (b) luminal subtype breast cancer patients alone, (c) triple-negative breast cancer patients alone.

Although adjuvant therapies are effective in breast cancers, their employment was not yet standard practice in Japan in the 1980's. Here, we first compared the outcome for breast cancer patients treated at Nagoya City University Hospital in the 1980's with those treated in the 1990's. All patients treated later tended to show better DDFS compared with those treated in the 1980's (Fig. 3a). Patients with tumors of luminal subtype treated in the 1990's showed significantly better DDFS than those from the 1980's (P = 0.02, Fig. 3b). In contrast, DDFS of patients with TNBC treated either in the 1980's or 1990's was similar (Fig. 3c). Only 58% (61/106 cases) of patients with luminal subtype tumors treated in the 1980's received adjuvant tamoxifen therapy while this figure increased to 78% (194/248 cases) in the 1990's. Therefore, a higher rate of adjuvant hormone therapy might be associated with better DDFS in patients with luminal subtype tumors. For TNBCs, 31% (12/39 cases) treated in the 1980's received chemotherapy (10 patients received oral 5-fluorouracil (5-FU), and 2 were treated with an anthracycline-based regimen) while 38% (12/32 cases) received chemotherapy in the 1990's (11 oral 5-FU, and one CMF (cyclophosphamide/methotrexate/5-FU)-based regimen). Because both the percentage of patients on adjuvant therapy and the adjuvant chemotherapy regimens themselves were similar for TNBC patients in the 1980's and the 1990's, the outcomes in these two treatment epochs were considered to be similar.

### Clinical outcome according to tumor subtype

**Figure 4:**
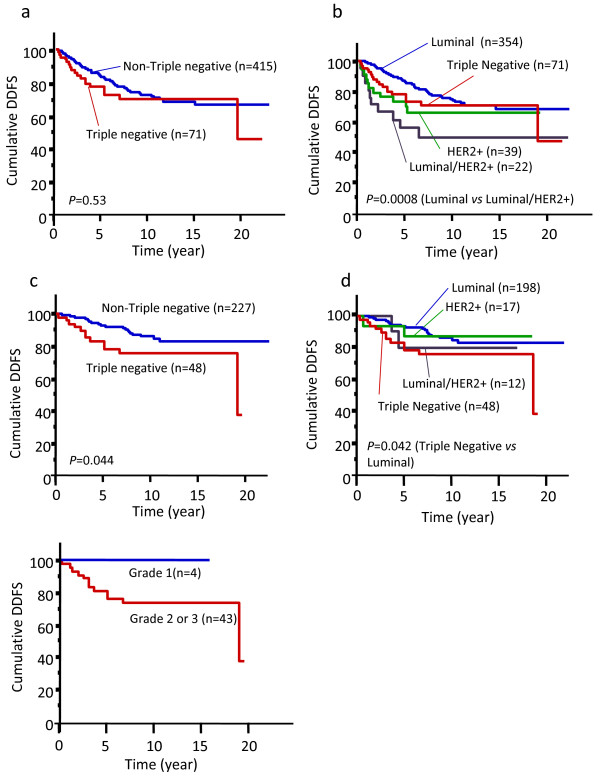
**Distant disease-free survival (DDFS) according to breast cancer subtype.** (a)(b) all patients, (c)(d) node-negative patients alone, (e) node-negative triple-negative breast cancer patients alone.

Next, we analyzed the outcome of all patients treated from 1981 to 1999 (n = 531) to compare TNBCs with non-TNBCs. We found that TNBC was not associated with poor DDFS overall (Fig. 4a). Non-TNBC were classified into 3 subtypes as follows: 1) luminal subtype (ER+ and/or PgR+ and HER2-), 2) luminal/HER2+ subtype (ER+ and/or PgR+ and HER2+), and 3) HER2+ subtype (ER- and PgR- and HER2+) [3]. Within 5 years of diagnosis, the Kaplan-Meier survival curves of TNBC and HER2+ subtype patients were similar (Fig. 4b). Thereafter, however, the survival curve of TNBCs more closely approximated luminal subtype. DDFS was significantly different between luminal and luminal/HER2+ subtypes (P = 0.0008) but not between TNBC and any other subtype (Fig. 4b). For luminal/HER2+ subtype patients, 6 (27%) received no adjuvant therapy and 7 (32%) received adjuvant tamoxifen alone. Therefore, patients with luminal/HER2+ subtype tumor could be considered to have been under-treated in this series.

In our series, 68% of TNBCs were node-negative but only 53% of non-TNBCs (P = 0.02, Table 1). Therefore, we next analyzed the clinical outcome only in node-negative breast cancers. Fifteen patients (31%) having node-negative TNBC received adjuvant chemotherapy (13 oral 5-FU, and 2 anthracycline-based regimen) while 26 (54%) received no adjuvant therapy. Medical records of 7 patients (15%) having node-negative TNBC were missing. The results show that node-negative TNBC was associated with shorter DDFS than node-negative non-TNBC (Fig. 4c). When DDFS of node-negative TNBCs was compared with other subtypes, a significant difference was observed only between TNBC and luminal subtype (Fig. 4d). As shown in Fig. 4e, grade 1 node-negative TNBCs demonstrated no distant metastasis. However, the number of these patients was very small. Univariate Cox regression analysis indicated that only nodal status was associated with DDFS in TNBCs (Table 5).

**Table 5:** Univariate Cox Regression Analysis of Factors Associated with Distant Disease-free Survival in the Triple-Negative Tumors

Variables	P value	Relative Risk of recurrence (95% CI)
Size		
≤2 cm		1.0 (reference)
>2 cm	0.92	1.26 (0.44 to 3.65)
Nodal status		
Negative		1.0 (reference)
Positive	0.02	2.60 (1.14 to 6.96)
Grade		
1		1.0 (reference)
2, 3	0.22	2.46 (0.68 to 10.39)
TP53 expression		
Negative		1.0 (reference)
Positive	0.61	1.12 (0.72 to 1.77)
Ki67 expression		
Low		1.0 (reference)
High	0.55	0.88 (1.59 to 1.34)
EGFR IHC		
Negative		1.0 (reference)
Positive	0.88	1.07 (0.48 to 2.36)
*EGFR *gene copy number		
<3		1.0 (reference)
≥3	0.2	0.27 (0.03 to 2.05)
Cytokeratins		
Negative		1.0 (reference)
Positive	0.42	0.72 (0.33 to 1.68)
Basal like type		
+		1.0 (reference)
-	0.67	0.79 (0.34 to 1.80)

IHC, immunohistochemistry

## Discussion

Although adjuvant hormone therapy was shown to be effective for ER-positive breast cancers [25], and adjuvant trastuzumab therapy was also shown to improve the survival of HER2 positive breast cancers [26,27], TNBCs have no targeted therapy at present. We screened TNBCs for 14 known *EGF*R mutations previously reported in lung cancers [18] but failed to find any. Gefitinib was reported to be effective not only in lung cancers with *EGFR *mutations [23,24,28], but also for lung or colorectal cancers expressing EGFR protein or with increased *EGFR *gene copy number [20,29]. In our study, 31% of TNBCs were found to express EGFR protein, so could be candidates for gefitinib therapy. We also analyzed *EGFR *gene copy numbers of TNBCs by the PCR method [19-21], and found frequent increases in TNBCs compared with luminal subtype breast cancers. Although lung cancers having *EGFR *mutations were reported to be responsive to gefitinib therapy, about 10% of lung cancers without *EGFR *mutations were also susceptible to this therapy [28]. Therefore, even though no *EGFR *mutations were found in TNBCs in this study, TNBCs with increased *EGFR *gene copy number might still be candidates for therapy with anti-EGFR monoclonal antibodies such as gefitinib. We performed FISH assays on representative tumors with increased *EGFR *copy number [30] but could not compare the PCR and FISH assays. The reason for this was most likely the condition of the paraffin-embedded tissues not being good enough, because we did not see either *EGFR *or *CEP7 *signals as a control (data not shown). In our study, EGFR protein expression was not consistently accompanied by increased *EGFR *gene copy number. One of the reasons for lack of correlation between *EGFR *gene copy number and immunohistochemistry could be the technique used in this study. EGFR protein expression is generally heterogeneous. If a frozen tissue with PCR technique is used, it is subjected to the "dilution effect" and could be responsible for non-correlation in at least some cases. In lung cancers, increased *EGFR *gene copy number was also reported not to be consistently accompanied by positivity for EGFR protein, as assessed by immunohistochemistry [32]. Although we did not obtain FISH data for TNBCs with increased *EGFR *gene copy numbers, we are confident that the *EGFR *gene copy number data are reliable because the methodology to measure this gene is well-established [19-21], and in this series a significant difference in *EGFR *gene copy number was observed between TNBCs and luminal subtype tumors.

Basal-like breast cancer was reported to frequently possess *BRCA1 *germline mutations [2], and *BRCA1 *mRNA expression was also shown to be lower in sporadic basal-like breast cancers [8]. The profound similarities between hereditary *BRCA1*-related breast cancers and basal-like breast cancers suggest a fundamental defect in the *BRCA*1 pathway in sporadic basal-like breast cancers [33,34]. Recently, BRCA1 has been demonstrated to play a critical role in the differentiation of ER-negative stem/progenitor cells to ER-positive luminal cells, and it was reported that loss of BRCA1 function resulted in the accumulation of ER-negative stem/progenitor cells, candidates for a cancer stem cell in the basal-like subtype [35]. BRCA1 was demonstrated to have an important role for DNA double-strand break repair by homologous recombination [36]. Poly(ADP-ribose) polymerase (PARP) is an enzyme involved in base excision repair, a key pathway in the repair of DNA single-strand breaks [37]. Recently, BRCA1/2 dysfunction has been shown to sensitize cells to the inhibition of PARP enzymatic activity [38]. Therefore, PARP inhibitors may be key drugs for treating TNBCs [38].

Although patients with TNBCs were reported to have a poor prognosis [6,39], the recurrent pattern of TNBCs was different from non-TNBCs [40-42]. Dent et al [40] reported that TNBC patients had an increased likelihood of recurrence and death within 5 years of diagnosis but not thereafter, while the recurrent risk among non-TNBCs was shown to be stable over the period of follow-up. Liedtke et al [41] described the clinical course of 1,118 breast cancer patients who received neoadjuvant chemotherapy. Twenty-three percent of these women had TNBC, and showed an increased likelihood of 3-year progression-free survival and 3-year overall survival [41]. The clinical course of TNBCs in our series was very similar to these previous reports [40-42].

Patients with TNBC were reported to have increased pathological complete response rates (pCR) to neoadjuvant chemotherapy compared with non-TNBC [41,43,44]. However, TNBC patients with residual disease after neoadjuvant chemotherapy had worse survival compared with non-TNBC patients [41,43]. Carey et al [43] termed this characteristic of TNBCs "the triple negative paradox". The higher sensitivity to neoadjuvant chemotherapy in TNBCs may suggest that patients with TNBC should be treated more intensively including with novel therapeutic alternatives. In this series, node-negative TNBCs showed poorer prognosis than node-negative non-TNBCs. Node-negative TNBCs could thus be considered to have been under-treated with adjuvant therapy in this series. On the other hand, no recurrence occurred in grade 1 node-negative TNBCs, although this subgroup was very small. This might indicate another issue as to how to classify TNBCs into subgroups that would benefit from more intensive adjuvant therapy. In this study, we did not find any useful prognostic markers of TNBCs although they showed higher positivity for TP53 protein accumulation and higher Ki67 expression.

## Conclusion

Frequently increased *EGFR *copy number and EGFR protein expression, and decreased *BRCA1 *mRNA expression, were observed in Japanese triple-negative breast cancers. *EGFR *and *BRCA1 *might be candidates for targeted therapies.

## Competing interests

The authors declare that they have no competing interests.

## Authors' contributions

TT conceived and designed the study and drafted the manuscript. NK carried out mRNA expression assay. TT, HY and ST carried out immunohistochemical analysis. KO and HS carried out mutation and amplification assays. HI and YF were involved in data acquisition and interpretation. All authors read and approved the final manuscript.

## Pre-publication history

The pre-publication history for this paper can be accessed here:


